# Research on the influence mechanism of structural separation on the damage excitation of bridge piers under seismic motion

**DOI:** 10.1371/journal.pone.0342310

**Published:** 2026-02-25

**Authors:** Yuwen Wen, Yang Liu, Xiaojuan Ning, Houzheng Xia, Wenjun An

**Affiliations:** Jiangxi University of Engineering, School of Civil Engineering, Jiangxi, China; Tongji University, CHINA

## Abstract

This study builds a special numerical model based on the OpenSees platform, aiming to explore the influence of structural separation on the damage of bridge piers under excitation. The key parameters and threshold conditions related to structural separation were clarified by using simple harmonic excitation. The validity of the established theoretical framework was verified through multiple actual seismic waves, as well as the effect of vertical separation of piers and beams on damage development. At the same time, the damage differences of piers at different heights were compared in combination with vulnerability analysis, with a focus on the regulatory effect of separation on pier failure. The research results show that there is a significant correlation between the excitation parameters and the structural response. Especially when the seismic period approaches the first-order vertical natural vibration period of the bridge, the maximum axial pressure of the bearing increases exponentially with the amplitude, and after the separation occurs, the sensitivity of the collision force to the amplitude is significantly reduced. The working state of bridge piers is significantly affected by seismic waves and the connection form of the bearing type, which leads to regular changes in the structural safety state and failure mode due to the differences in seismic wave characteristics. Meanwhile, the height of the bridge pier plays a key role in the structural response. As the height of the bridge pier increases, the maximum contact force shows a downward trend. However, the vertical separation of the pier and the beam is more likely to cause severe damage or even complete failure of the bridge pier. The above research conclusions provide theoretical references of practical significance for strengthening the seismic design of Bridges.

## 1 Introduction

The influence of vertical ground motion on the seismic performance of bridge structures is significant and often underestimated. Together with long-period pulse effects, it constitutes the core mechanism by which near-fault ground motion damages Bridges [1]. The probability that the ratio of the vertical to horizontal peak acceleration in the near-fault area exceeds the 2/3 limit of the specification is 70%, and in extreme cases, this ratio can reach 1.82 [2]. This high-amplitude vertical excitation will increase the axial force fluctuation range of the bridge pier by 87%, triggering alternating tensile and compressive stresses and reducing the shear resistance [3]. It will also transform the failure mode from compression bending to brittle shear failure, increasing the curvature ductility coefficient at the bottom of the bridge pier by 70% [4], and at the same time lead to beam-pier separation and an 80% increase in the residual displacement Angle of the bearing [5]. When the horizontal and vertical pulses overlap (accounting for 35% of the near-fault records), the structural damage entropy value is 2.1 times that of a single horizontal excitation [6].

Existing research has verified its influence from multiple dimensions: Shid et al. ‘s analysis of 40 seismic records through IDA technology indicates that the vertical ground movement direction has a significant impact on the FPS system [7]; Tamaddon et al. found that in the epicenter near the source, non-uniform vertical excitation would intensify the lateral displacement of the concrete piers of the curved box girder, and the change in the excitation frequency of the piers might increase the impact force by three times [8,9]; When the V/H ratio in the medium and high seismic activity zone exceeds 1, the vertical ground movement has a significant impact on the deformation and internal forces of the upper and lower structures of the curved skew bridge, and its response is more complex than that of the straight bridge [10]. Research on multi-span continuous steel-concrete composite box girders reveals that the prestress level has a significant impact on the elastic stage, strain distribution and crack resistance of the structure. Moreover, the prestress state under vertical ground motion further amplifies this influence. The optimal prestress scheme can enhance the critical and ultimate loads of the structure’s elastic-plastic properties [11]. The seismic performance prediction framework of the vehicle-bridge interaction (VBI) system based on LGBM shows that seismic intensity parameters and bridge pier height have a significant impact on the structural response under vertical ground motion, providing an efficient tool for quantifying the effect of vertical ground motion [12]. At present, it is urgently necessary to correct the estimation deviation of vertical components in the specification and establish an analysis framework for pulse-vertical coupling effects [13].

**Fig 1:**
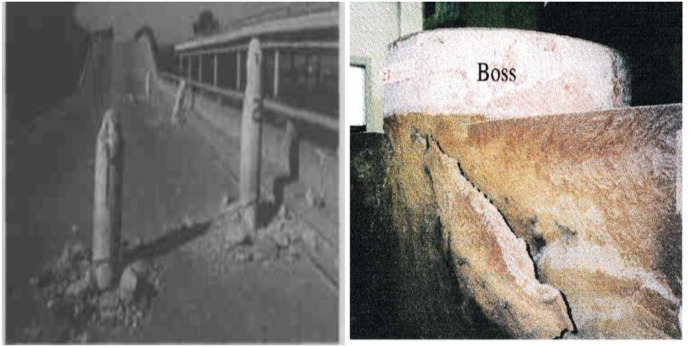
Bridge damage caused by earthquakes.

The vertical components of near-fault pulse-type ground motion can significantly increase the possibility of Bridges exceeding the damage threshold [14]. The physical seismic model constructed for the southern seismic belt of Iceland, after being calibrated by Bayesian inference, can accurately simulate the temporal history of strong earthquakes and the characteristics of near faults, providing a precise simulation tool for revealing the trigger timing and intensity threshold of structural separation phenomena [15]; The study of the focal depth in the central Indian tectonic belt found that the focal depth was unevenly distributed within the range of 5–38 kilometers [16]. The earthquake disaster assessment study in Noida, India, identified the F1 fault as the main focal point, and the analysis of its related ground motion parameters provided a reference for clarifying the trigger focal conditions of structural separation in specific fault areas [17]. The rubber bearings commonly used in China belong to the “weak connection” structure. Under the vertical seismic excitation, the main beam is prone to significant deformation, which in turn leads to the separation of the main beam from the bearing [1]. Tanimura believes that the direct cause of the fracture of the Nielson bridge bearing is the vertical collision between the main beam and the pier [18,19], and the structural separation in the decoupled state will amplify the horizontal dynamic response of the pier and increase its risk of damage [20,21], refer to the schematic diagram in Fig 1.

The vertical separation coupled pier (VSCP) system proposed by Wang et al. indirectly reduces the synergistic damage between the bearing and the pier by expanding the effective contact area, reducing concrete spalling and stress concentration [22]. Liu et al. found that the shock absorption effect of QZS and vertical dampers depends on key parameters such as the eccentricity of the isolation system [23]; Research on the isolation system of large substructures shows that the isolation effect is best when the horizontal damping ratio is 0.3 and the vertical damping ratio is 0.2 [14,24]. After being calibrated by Bayesian regression and MCMC simulation, the Icelandic ground motion model is unbiased and has a total standard deviation of approximately 0.17 [25]. The precise ground motion parameters it provides can offer data support for the selection of support types.

**Fig 2:**
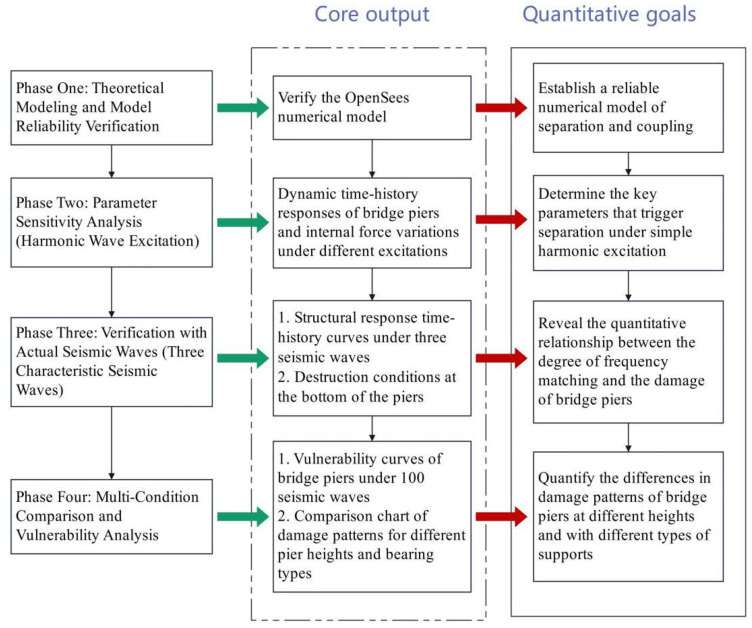
Technical Roadmap.

The height of bridge piers is a key parameter affecting the response of Bridges under near-fault ground motion, and its regulatory effect is reflected in two aspects: dynamic response characteristics and damage degree. The M-Usami model proposed by Lin et al. is more effective in predicting the damage degree of high-pier concrete bridge piers under bidirectional seismic forces, and energy dissipation has a significant impact on the damage degree [26]. Tubaldi et al. emphasized the influence of axial force on the shear force and bending moment response of high pier foundations, highlighting the crucial role of high-order modes in the distribution of shear force and bending moment [27]. Comparative studies on bridge piers of different heights show that the influence of bearing types on the damage of bridge piers is highly dependent [1], while the adoption of the optimized 3D-TMD can effectively reduce the peak bending moment response of high piers [14]. The research on the seismic performance prediction of the VBI system directly confirms that the height of bridge piers has a significant impact on the seismic response of Bridges, and together with the seismic strength parameters, they constitute the core influencing factors [12];

This study aims to systematically explore the influence mechanism of structural separation phenomenon on the damage excitation of bridge piers. Relying on the OpenSees platform, a numerical analysis model is established, and a phased progressive research method is adopted: first, parameter sensitivity analysis is carried out using simple harmonic excitation to clarify the key excitation parameters and trigger conditions, as well as the influence threshold related to structural separation behavior; Then, actual seismic waves with different characteristics are introduced as input to verify the theoretical analysis framework and quantify the influence of structural separation on the damage process of bridge piers. Finally, compare the calculation data of bridge piers of different heights under tensile and non-tensile bearing conditions to clarify whether the influence of bearing type on the damage excitation of bridge piers shows a dependent characteristic with the height of the piers. The specific technical roadmap is shown in Fig 2.

## 2 Bridge model and theoretical solution

### 2.1 Theoretical model

This study presents a simplified theoretical model for the structural analysis of a four-span continuous girder bridge, aiming to effectively capture the force-transfer behavior of its critical components. The model consists of three primary components: the main girder is represented by an Euler-Bernoulli beam model, which neglects shear deformation to focus on the flexural deformation behavior of the structure, making it particularly suitable for slender beam elements; the piers are modeled using the Saint-Venant bar model, which highlights axial and bending deformations to simulate the overall mechanical response of the piers under loading; and the bearings are characterized by spring models that reflect the stiffness properties, as well as the restraints and deformation capacities imposed by the bearings on the superstructure in both vertical and horizontal directions.

To verify the applicability of the Euler-Bernoulli beam model (i.e., neglecting shear deformation) in this study, the slenderness ratio of the main girder was evaluated. The calculated slenderness ratio L/h = 19 (see Chapter 3 for parameters), which exceeds the commonly used critical value of 15 in bridge engineering design (when the beam element length is more than 15 times a characteristic cross-section dimension, it is generally considered a slender beam and shear deformation may be neglected). The results indicate that the main girder in this study is a slender member for which shear deformation effects are negligible; therefore, the chosen theoretical model is appropriate and consistent with the analysis objectives.

For the piers, a Saint-Venant rod model that considers only axial deformation was employed, thereby disregarding the element’s torsional degrees of freedom. The rationale for this simplification rests on two key points: first, the focus of this study is a straight beam bridge with symmetric structural and mass distributions about the longitudinal axis, which results in minimal torsional response under vertical seismic excitation; second, in the context of near-fault vertical ground motions, the input energy predominantly manifests as axial tension-compression excitation, rendering the torsional excitation component negligible. Since this study emphasizes the axial and flexural responses of the piers, and torsional effects are not the primary controlling factors, this simplified model is theoretically justified and aligns with conventional analytical practices for such bridges. Supporting literature (reference [a]) further validates this simplification; that work compared solutions derived from the theoretical approach utilized here with APDL finite element solutions, demonstrating that for relatively short piers (H < 18 m), harmonic excitation can sufficiently replicate the dynamic response of the bridge, thereby reinforcing the applicability of the current model within the specified parameter range.

The main beam comprises two segments connected by hinged joints, facilitating relative rotation between adjacent segments. This connection type releases bending moments, transmitting solely vertical shear and horizontal forces. The pier base is rigidly connected, establishing a firm link with the foundation. This rigid connection restricts pier base rotation and movement, thereby enhancing structural stability.

**Fig 3:**
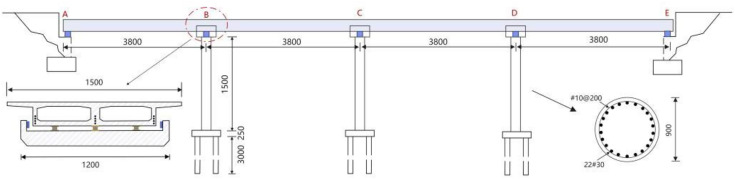
Theoretical model of the bridge.

The bridge is subjected to vertical seismic excitation through the uniform excitation method to analyze dynamic load effects. This method assumes that seismic waves have consistent input across the entire bridge structure, neglecting spatial variations during propagation. This simplifies the dynamic response analysis under seismic action, as depicted in Fig 3.

Under vertical excitation, the displacement equations for the girder and pier are given as follows:


$$\begin{array}{cc} \rho_{b}A_{b}\frac{\partial^{\text{2}}Y_{i}(x,t)}{\partial t^{\text{2}}}-\frac{\partial^{\text{2}}(E_{b}I_{b}\partial^{\text{2}}Y_{i}(x,t)/\partial x^{\text{2}})}{\partial x^{\text{2}}}+q=0 & \left(i=1,2,3,4\right) \end{array}$$ (1)

$$\begin{array}{cc} E_{r}A_{r}\frac{\partial^{\text{2}}u_{j}\left(\xi ,t\right)}{\partial^{\text{2}}\xi }-\rho_{r}A_{r}\frac{\partial^{\text{2}}u_{j}\left(\xi ,t\right)}{\partial t^{\text{2}}}=0 & \left(j=1,2,3\right) \end{array}$$ (2)

In these equations, *Y* denotes the vertical displacement of the girder, and *U* represents the vertical displacement of the pier. The variable *q* stands for the dead weight of the main girder, while *x* and ξ are the vertical coordinates of the girder and pier, respectively. Here, *i* indicates the number of the main girder in the model, and *j* denotes the pier number, with specific numbering detailed in Fig 1. The definitions of *i* and *j* remain consistent in subsequent equations.


$$\begin{array}{cc} Y_{i}\left(x,t\right)=Y_{is}\left(x,t\right)+Y_{ig}\left(x,t\right)+Y_{id}\left(x,t\right) & \left(i=1,2,3,4\right) \end{array}$$ (3)

$$\begin{array}{cc} U_{j}\left(\xi ,t\right)=U_{js}\left(\xi ,t\right)+U_{jg}\left(\xi ,t\right)+U_{jd}\left(\xi ,t\right) & \left(j=1,2,3\right) \end{array}$$ (4)

In the above formulas, the subscript *s* refers to static deflection deformation, *g* represents overall rigid-body displacement, and d denotes the dynamic deformation of the structure.

### 2.2 Solution to the bridge motion equation

The static deformation of the main girder during the contact stage is expressed as:


$$\begin{array}{cc} Y_{s1}(x)=\frac{\text{1}}{\text{3}}F_{z}(2L+x{)}^{\text{3}}-\frac{\text{1}}{\text{8}}q(2L+x{)}^{\text{4}} & -2L\leq x<-L\\ Y_{s2}(x)=\frac{\text{1}}{\text{3}}[F_{z}(2L+x{)}^{\text{3}}+F_{b}(L+x{)}^{\text{3}}]-\frac{\text{1}}{\text{8}}q(2L+x{)}^{\text{4}} & -L\leq x<0 \end{array}$$ (5)

Where $F_{z}$ is the support reaction at point B, and $F_{b}$ is the support reaction at point C, both calculated via the deformation coordination equation. Utilizing symmetry, the static deflection deformations $Y_{s3}$ and $Y_{s4}$ are derived.

The static deflection deformation of the pier is:


$$\begin{array}{cc} U_{s1}\left(\xi \right)=U_{s3}\left(\xi \right)=\frac{F_{z}\xi }{E_{r}A_{r}} & U_{s2}\left(\xi \right)=\frac{F_{b}\xi }{E_{r}A_{r}} \end{array}$$ (6)

The vertical rigid-body displacement of the bridge during the contact stage is given by:


$$\begin{array}{cc} Y_{ig}\left(x,t\right)=U_{jg}\left(\xi ,t\right)=B\left(t\right) & \left(i=1,2,3,4;\;j=1,2,3\right) \end{array}$$ (7)

By substituting Equations (3) and (4) into Equation (2), the wave equations governing the dynamic displacements $Y_{id}$ and $U_{id}$ of the bridge can be solved:


$$\begin{array}{cc} \frac{\partial^{\text{2}}\left(E_{b}I_{b}\frac{\partial^{\text{2}}Y_{id}\left(x,t\right)}{\partial x^{\text{2}}}\right)}{\partial x^{\text{2}}}+\rho_{b}A_{b}\frac{\partial^{\text{2}}Y_{id}\left(x,t\right)}{\partial t^{\text{2}}}=-\rho_{b}A_{b}\frac{\partial^{\text{2}}Y_{ig}\left(x,t\right)}{\partial t^{\text{2}}} & \left(i=1,2,3,4\right) \end{array}$$ (8)

$$\begin{array}{cc} E_{r}A_{r}\frac{\partial^{\text{2}}U_{jd}\left(\xi ,t\right)}{\partial^{\text{2}}\xi }+\rho_{r}A_{r}\frac{\partial^{\text{2}}U_{jd}\left(\xi ,t\right)}{\partial t^{\text{2}}}=-\rho_{r}A_{r}\frac{\partial^{\text{2}}U_{jg}\left(\xi ,t\right)}{\partial t^{\text{2}}} & \left(j=1,2,3\right) \end{array}$$ (9)

The dynamic deformation of the bridge is computed using the transient wave function expansion method, which involves superimposing a series of wave mode functions and time functions. The specific formulas are as follows:


$$\begin{array}{cc} Y_{id}\left(x,t\right)=\sum_{n=1}^{\infty }\varphi_{nbi}\left(x\right)q_{n}\left(t\right) & \left(i=1,2,3,4\right) \end{array}$$ (10)

$$\begin{array}{cc} U_{jd}\left(x,t\right)=\sum_{n=1}^{\infty }\varphi_{nrj}\left(x\right)q_{n}\left(t\right) & \left(j=1,2,3\right) \end{array}$$ (11)

Among them, $\varphi_{nbi}$ represents the wave function equation of the main girder, and $\varphi_{nrj}$ represents the wave function equation of the bridge pier, $while\;q_{n}(t)$ denotes the temporal function during the contact stage.

The wave functions for the girder and pier are:


$$\varphi_{nbi}=A_{ni}sink_{bn}x+B_{ni}cosk_{bn}x+C_{ni}sinhk_{bn}x+D_{ni}coshk_{bn}x\text{(i=1,2,3,4)}$$ (12)

$$\begin{array}{cc} \varphi_{nrj}=E_{nj}\;sin\;k_{rn}\xi +F_{nj}\;cos\;k_{rn}\xi & \text{(j=1,2,3)} \end{array}$$ (13)

In these formulas, $A_{ni},B_{ni},C_{ni},D_{ni}$ are the coefficients of the girder’s bending wave functions, $E_{nj},F_{nj}$ are the coefficients of the pier’s axial wave functions, and $k_{bn}$ and $k_{rn}$ respectively represent the wave numbers of the main beam and the pier.

The dynamic displacement boundary conditions of the calculation model are:


$$\begin{array}{c} \varphi_{nb1}(-2L)=\varphi_{nb1}^{\prime \prime }(-2L)=\varphi_{nb4}\left(2L\right)=\varphi_{nb4}^{\prime \prime }\left(2L\right)=0\\ \varphi_{nr1}\left(0\right)=\varphi_{nr1}^{\prime }\left(0\right)=\varphi_{nr2}\left(0\right)=\varphi_{nr2}^{\prime }\left(0\right)=\varphi_{nr3}\left(0\right)=\varphi_{nr3}^{\prime }\left(0\right)=0 \end{array}$$ (14)

The continuity conditions for displacement and force at the model’s connection are:


$$\begin{array}{c} \varphi_{nbi}(-2L+iL)=\varphi_{nb(i+1)}(-2L+iL),\;\varphi_{nbi}^{\prime }(-2L+iL)=\varphi_{nb(i+1)}^{\prime }(-2L+iL)\\ {\varphi_{nbi}}^{\prime \prime }(-2L+iL)={\varphi^{\prime \prime }\;\;\;}_{nb(i+1)}(-2L+iL),\;\varphi_{nbi}(-2L+iL)=\varphi_{nri}\left(H\right)+\frac{E_{r}A_{r}\varphi_{nri}^{\prime }\left(H\right)}{K_{c}} \end{array}$$ (15)

$$E_{b}I_{b}k_{bn}^{\text{3}}\left[\varphi_{nbi}^{\prime \prime \prime }(-2L+iL)-\varphi_{nbi+1}^{\prime \prime \prime }(-2L+iL)\right]=E_{r}A_{r}\varphi_{nri}^{\prime }\left(H\right)$$ (16)

In Formulas (15) and (16), i = 1, 2, 3.

Substituting equations 14, 15 into equations 12, 13, the coefficients of the wave function equation can be obtained.

Taking point B as an example, through calculation, it can be concluded that:


$$\begin{array}{c} \varphi_{nb1}(-L)=\varphi_{nb2}(-L);\;\varphi_{nb1}^{\prime }(-L)=\varphi_{nb2}^{\prime }(-L);\;\varphi_{nb1}^{\prime \prime }(-L)=\varphi_{nb2}^{\prime \prime }(-L);\;\varphi_{nr1}\left(0\right)=0\\ \varphi_{nb1}(-2L)=\varphi_{nb1}^{\prime \prime }(-2L)=0;\;\varphi_{nb1}(-2L)=\varphi_{nr1}\left(H\right)+\frac{E_{r}A_{r}\varphi_{nr1}^{\prime }\left(H\right)}{K_{c}} \end{array}$$ (17)

It can be concluded that:


$$\begin{array}{c} \varphi_{nbi}\left(x\right)=A_{n1}(sin\;k_{bn}x-tan\;k_{bn}L\;cos\;k_{bn}x)+C_{n1}(sinh\;k_{bn}x-tanh\;k_{bn}L\;cosh\;k_{bn}x)\\ \varphi_{nr1}\left(\xi \right)=E_{n1}\;sin\;k_{rn}\xi \end{array}$$ (18)

Among them, when *i*=1, x∈[−2L,−L], and *i*=2, x∈[−L,0].

Similarly, the wave mode functions of the remaining main beams and piers can be calculated.

The wave functions of the bridge structure exhibit orthogonality and normalization. For distinct eigenvalues *m* and *n*, the characteristic functions of the girder and rod satisfy the following characteristic equations:


$$\begin{array}{c} {a_{\text{1}}}^{\text{2}}\varphi_{mb\text{i}}^{\left(4\right)}-\omega_{m}^{\text{2}}\varphi_{mbi}=0\text{\textbackslash{}hspace\{0.33em}\;\;\;\;\;\;\;\;\;\;\}{a_{\text{1}}}^{\text{2}}\varphi_{nbi}^{\left(4\right)}-\omega_{n}^{\text{2}}\varphi_{nbi}=0\\ {c_{\text{1}}}^{\text{2}}\varphi_{mrj}^{\left(2\right)}+\omega_{m}^{\text{2}}\varphi_{mrj}=0\text{\textbackslash{}hspace\{0.33em}\;\;\;\;\;\;\;\;\;\;\}{c_{\text{1}}}^{\text{2}}\varphi_{nrj}^{\left(2\right)}+\omega_{n}^{\text{2}}\varphi_{nrj}=0 \end{array}$$ (19)

Multiply the main girder equations in the above set by $\varphi_{nbi}$ and $\varphi_{mbi}$, and the pier equations therein by $\varphi_{nrj}$ and $\varphi_{mrj}$. Then, subtract the two resulting sets of equations, perform integration, and finally sum them to obtain the following equation:


$$\text{\textbackslash{}hspace\{0.33em}\;\;\}(\omega_{m}^{\text{2}}-\omega_{n}^{\text{2}})[\sum_{i=1}^{\text{4}}\int_{-2S}^{-2S+iS}\rho_{b}A_{b}\varphi_{mbi}\left(x\right)\varphi_{nbi}\left(x\right)dx+\sum_{j=1}^{\text{3}}\int_{\text{0}}^{H}\rho_{r}A_{r}\varphi_{mrj}\left(\xi \right)\varphi_{nrj}\left(\xi \right)d\xi ]=0$$ (20)

From this, the conclusion is drawn that:


$$\sum_{i=1}^{\text{4}}\int_{-2S}^{-2S+iS}\rho_{b}A_{b}\varphi_{mbi}\left(x\right)\varphi_{nbi}\left(x\right)dx+\sum_{j=1}^{\text{3}}\int_{\text{0}}^{H}\rho_{r}A_{r}\varphi_{mrj}\left(\xi \right)\varphi_{nrj}\left(\xi \right)d\xi =\delta_{mn}$$ (21)

Where $\delta_{mn}$ is the Kronecker function.

Substituting formulas 16 and 19 into formula 18, the coefficients of the wave function and the vertical natural frequency can be obtained. It should be noted that Formula 18 only introduces the solution of the wave function at point B. The calculation methods for points C and D remain the same as those for point B.

The differential equation for the time function $q_{n}(t)$ of the bridge structure is:


$${\ddot{q}}_{n}\left(t\right)+\omega_{n}^{\text{2}}q_{n}\left(t\right)={\ddot{Q}}_{n}\left(t\right)$$ (22)

Where:


$$Q_{n}\left(t\right)=-\sum_{i=1}^{\text{4}}\int_{-3S+iS}^{-3S+iS}\rho_{b}A_{b}\varphi_{nbi}\left(x\right)Y_{ig}\left(x,t\right)dx-\sum_{j=1}^{\text{3}}\int_{\text{0}}^{H}\rho_{r}A_{r}\varphi_{nrj}\left(\xi \right)U_{jg}\left(\xi ,t\right)d\xi$$ (23)

Applying the Laplace transform to Formula 21 allows the determination of the bridge structure’s time function:


$$q_{n}\left(t\right)=q_{n}\left(0\right)cos\;\omega_{n}t+\frac{\text{1}}{\omega_{n}}{\dot{q}}_{n}\left(0\right)sin\;\omega_{n}t+\frac{\text{1}}{\omega_{n}}\int_{\text{0}}^{t}{\ddot{Q}}_{n}\left(\tau \right)sin\;\omega_{n}(t-\tau )d\tau$$ (24)

## 3 Finite element model of continuous beam bridge

### 3.1 Actual model of the bridge

**Fig 4:**
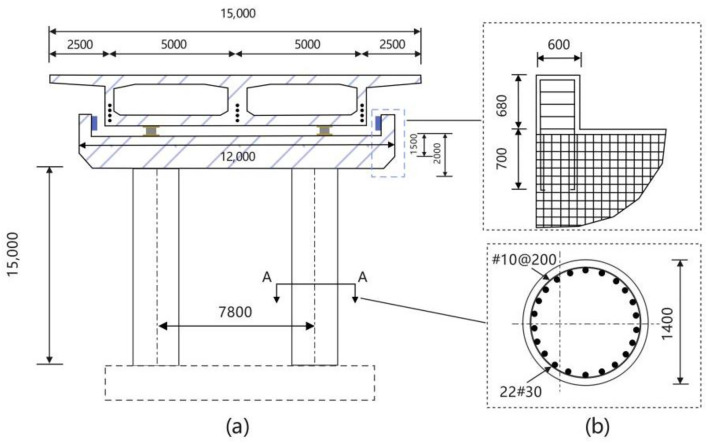
Cross-section view of the bridge.

This chapter discusses the influence of vertical excitation on the separation between bridge piers and girders, using a suitably sized two-span continuous box-girder bridge as the subject of analysis. In accordance with bridge construction practices in our country, the selected bridge structure is shown in Fig 4. The bridge piers consist of circular reinforced concrete columns with two helical hoop reinforcements and longitudinal steel bars./u

During the calculation process, for the convenience of analysis, the calculation parameters of the bridge model refer to the specification [28]. Plate rubber bearings are used between the main beam and the bridge piers. The specific parameter calculation results are shown in Table 1.

**Table 1:** Parameters of Main Beams and Piers.

Main beam parameters		Bridge pier parameters	
Single-span length	*L* = 38 m	Single-span length	*H* = 16 m
Density	*ρ* = 2600 kg/m^3^	Density	*ρ* = 2600 kg/m^3^
Equivalent cross-sectional area	*A*_*b*_ = 6.06m^2^	Equivalent cross-sectional area	*A*_*r*_ = 6.06m^2^
Moment of inertia of the equivalent section	*I*_*b*_ = 3.409m^4^	Moment of inertia of the equivalent section	*I*_*r*_ = 3.409m^4^
Equivalent elastic modulus	*E*_*b*_ = 34.5 GPa	Equivalent elastic modulus	*E*_*r*_ = 34.5 GPa

### 3.2 Finite element modeling

**Fig 5:**
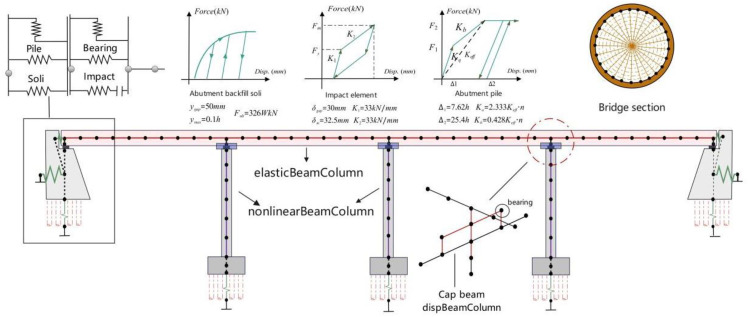
Finite element model of the bridge.

In this study, three sets of three-dimensional nonlinear finite element models were constructed using the open-source software OpenSees. Through a hierarchical and refined modeling strategy, an accurate simulation of the bridge structure has been achieved. Specifically, the box-shaped main beam is represented by an elasticBeamColumn, assuming that it maintains an elastic stress state under seismic action. The cap beams were modeled in detail using disposition-coordinated beam columns (disbeamcolumn), while the bridge piers adopted fiber beam columns (nonlinearBeamColumn), fully considering the nonlinear displacement response of the structure under seismic action. This fiber unit adopts a 7-point Gaussian integral scheme and fiber section discretization technology, which can effectively capture the nonlinear seismic response characteristics of bridge piers. The constitutive relationship of the surface layer concrete material adopts the KT-Park bilinear model (Concrete01). The parameters are set as follows: peak strength is 40 MPa, peak strain is 0.002, and ultimate strain is 0.004. Based on considering the constraint effect of the core concrete, its mechanical parameters were adjusted. The peak strength was 46 MPa, the peak strain was 0.0026, the residual strength was 32 MPa, and the ultimate strain was 0.008. The longitudinal reinforcing bars adopt the Menegotot-Pinto hysteresis model (Steel02), with a yield strength of 435 MPa. The yield strength of the transverse stirrup is 335 MPa. The elastic modulus of both the longitudinal and transverse reinforcing materials is 200 GPa, and the strain hardening ratio is 0.01 for both. In order to accurately characterize the pile-soil interaction effect at the structural interface, the contact element model proposed in the references was adopted to model the contact behavior among abutments, piles and backfill soil. The basic constraints adopt six-degree-of-freedom elastic bearings, combined with stiffness release in three translational and three rotational directions. This multi-scale modeling method effectively captures the mechanical response of bridge structures to seismic forces, providing a reliable numerical analysis framework for evaluating their seismic performance. The specific model is shown in Fig 5.

**Fig 6:**
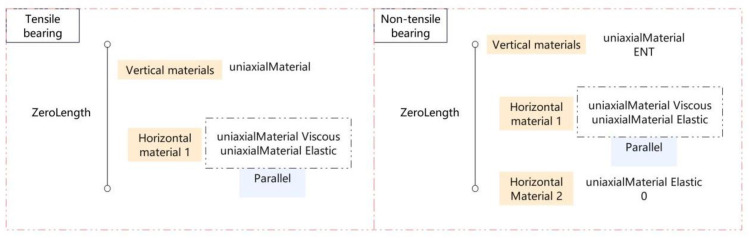
Bridge bearing element.

This study compares the impact of vertical separation of bridges on structural dynamic response by examining two types of bearings: non-tensile and tensile bearings. The simulation setup is illustrated in Fig 6. The vertical behavior of the tensile bearing is represented by a single elastic material, while the non-tensile bearing is modeled using an elastic no-tension (ENT) approach. Both bearings have a vertical elastic modulus of 1.0 GPa to accurately capture their compression characteristics. For horizontal behavior, the tensile bearing employs a parallel dual-material model: hysteretic energy dissipation is modeled by a viscous element, and linear elastic recovery is represented by a parallel elastic element. The shear elastic modulus in this direction is 1.0 MPa, with a damping ratio of 10% (referred to as Material 1).

**Fig 7:**
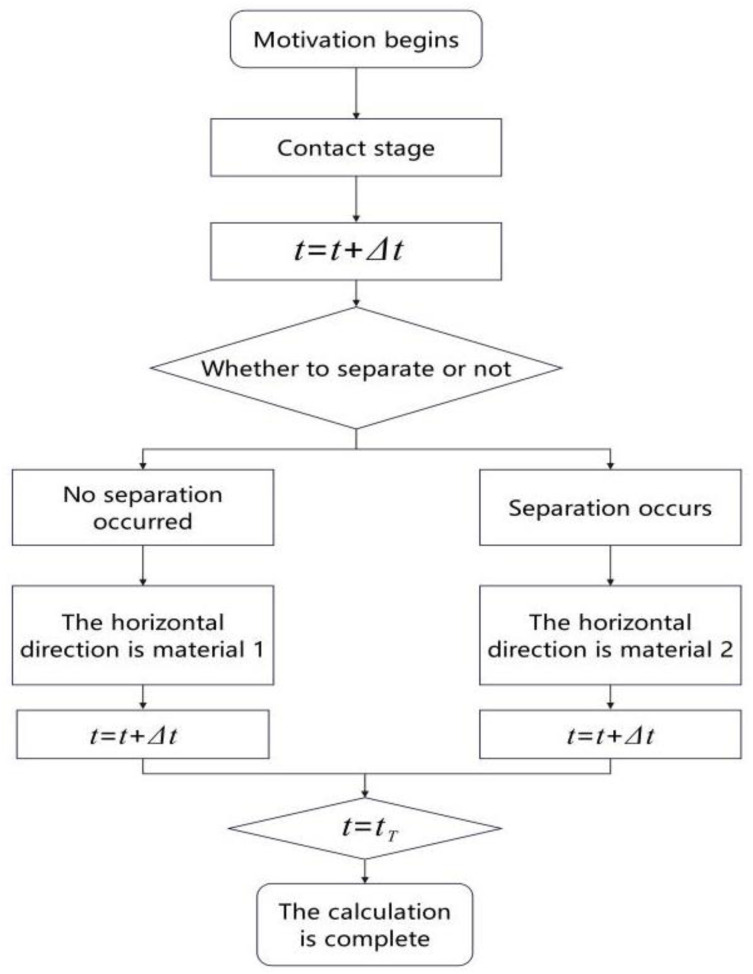
Flow chart of non-tensile support material switching.

In order to investigate the evolutionary behavior of the mechanical properties of non-tensile bearings under vertical separation conditions, a two-state material model is proposed. This model delineates the transition from Material 1 to Material 2 upon entering the separation state due to the loss of horizontal restraint, and the subsequent reversion to Material 1 with hysteresis elastic composite properties when the main girder reconnects with the bearing. The mechanical response curve depicted in Fig 7 distinctly illustrates the varying mechanical attributes of the bearing under contact and separation conditions, effectively elucidating the dynamic evolution of the state transition mechanism.

## 4 Dynamic response of bridges

### 4.1 Dynamic response verification

To validate the accuracy of the developed finite element model, a comparative assessment with theoretical solutions is imperative. To align the two approaches, specific simplifications are implemented in the finite element model: the main beam’s sections are linked via hinges, neglecting the abutment’s impact temporarily, and assuming a rigid connection of the pier’s base to the ground.

The seismic waves can be analyzed by decomposing them into a superposition of N simple harmonic waves using the fast Fourier transform. To validate the reliability of the theoretical model, simple harmonic wave excitation is utilized for verification purposes.

**Fig 8:**
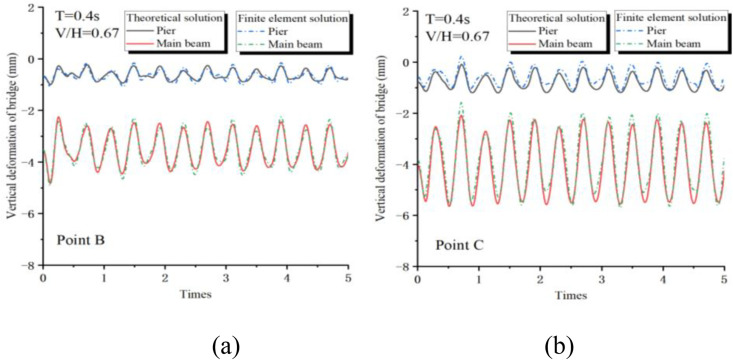
Bridge displacement response: (a) point B; (c) point C.

Fig 8 illustrates the comparison of vertical responses between the theoretical solution and the finite-element solution under operational conditions with a period of T = 0.4s and the ratio of vertical to horizontal acceleration of V/H = 0.67 (as per relevant specifications). Specifically, Fig 8a depicts the dynamic response curve at point B, while Fig 8b displays the dynamic response curve at point C. The comparison reveals a high degree of similarity between the finite-element solutions and the theoretical solutions at both locations. This similarity underscores the reliability of the finite-element model developed using OpenSees and its suitability for subsequent time-history analyses.

**Fig 9:**
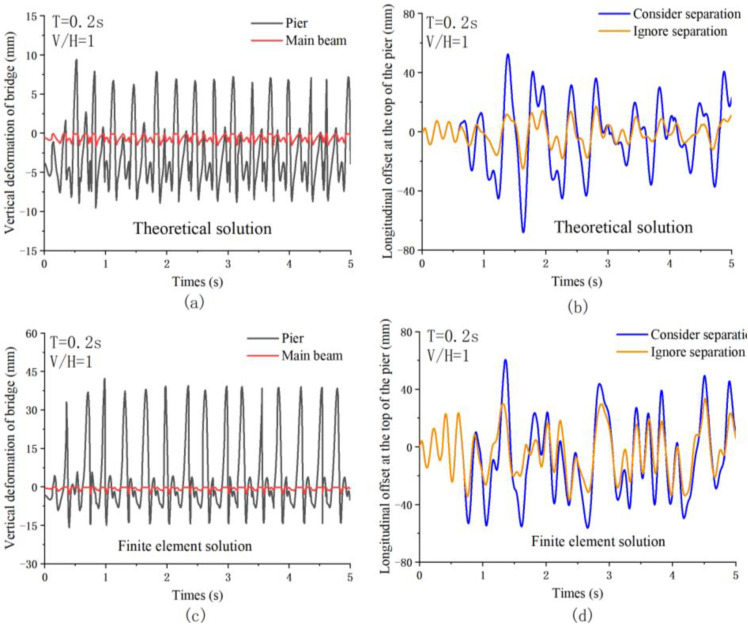
Horizontal dynamic responses of the bridge: (a) Pier No. 2; (b) Pier No. 1.

Given the heightened sensitivity of the dynamic response at point C of the multi-span two-bridge, Fig 9 illustrates the time-history response results for this point under simple harmonic excitation (T = 0.2s, V/H = 1). Fig 9a and 9b depict the vertical and horizontal responses derived from the theoretical solution, respectively, while Fig 9c and 9d present the vertical and horizontal responses obtained through the finite element solution. This data supports a comparative analysis of the two calculation methods. In terms of vertical response characteristics, the results from both the theoretical and finite element solutions generally align well. Both approaches identify the vertical separation-collision phenomenon between the main beam and the bridge pier; however, notable differences exist. The finite element solution reveals a more pronounced dynamic separation phenomenon of the bridge, resulting in a greater upward throw height of the main beam, which subsequently intensifies the collision effect between the main beam and the bridge pier.

The results of the longitudinal response analysis indicate that both calculation methods confirm that vertical separation significantly enhances the horizontal dynamic response of bridge piers. When the separation effect is disregarded, the theoretical solution calculates a peak longitudinal offset at the top of the bridge pier of 18.4 mm, which is less than the 32.6 mm observed in the finite element solution. The discrepancy arises primarily from two factors: First, the vertical impact force derived from the finite element analysis is greater, leading to a more pronounced effect on the horizontal offset of the bridge pier. Second, the theoretical solution fails to account for the coupling effect of horizontal and vertical forces, resulting in deviations in the calculated outcomes. After incorporating the separation effect, the theoretical solution’s extreme value of the longitudinal offset for the bridge pier increased from 18.4 mm to 65.6 mm, reflecting a rise of 256.5%. Similarly, the finite element solution showed an increase in the extreme value of the longitudinal offset from 32.65 mm to 63.47 mm, representing a 94.4% increase. These findings further substantiate the substantial impact of vertical separation on the horizontal dynamic response of the bridge pier.

### 4.2 Response analysis under different excitations

**Fig 10:**
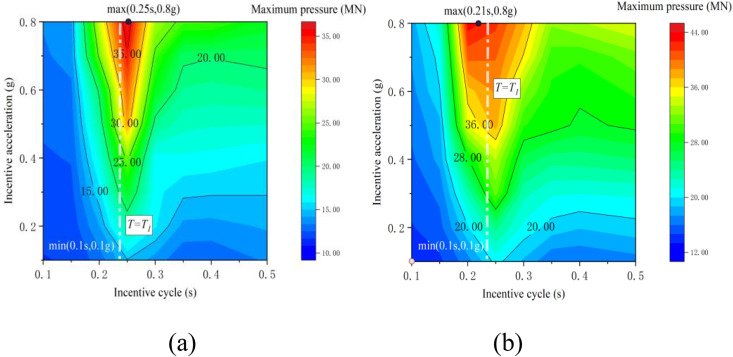
Cloud diagram of contact force: (a) point B; (c) point C.

Fig 10 illustrates contour maps depicting the vertical contact forces between the pier and the beam at points B and C as they vary with excitation period and amplitude. The numerical analysis findings suggest that when the earthquake period (T) approximates the first-order vertical natural vibration period of the bridge, the maximum axial pressure on the bearing increases exponentially with the earthquake amplitude. This increase significantly raises the likelihood of structural damage. During the non-separation phase, the peak axial pressure is directly proportional to the excitation amplitude. Upon entering the separation phase, the sensitivity of the collision force to the amplitude diminishes notably, with its growth rate significantly lower than that during the non-separation phase. This behavior arises from alterations in energy dissipation characteristics due to the nonlinearity of the contact interface. For continuous girder bridges with identical spans, the vertical collision response amplitude of the middle span surpasses that of the side spans.

**Fig 11:**
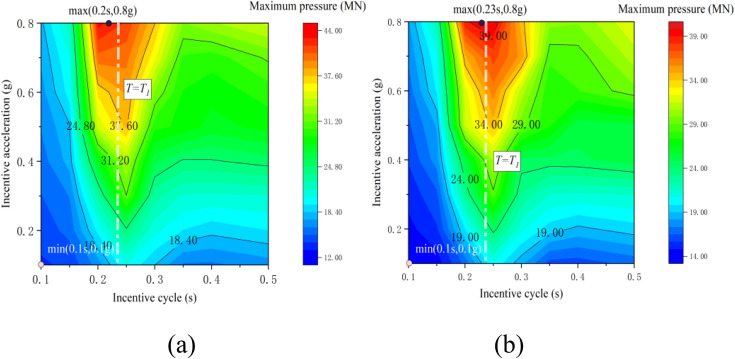
Cloud diagram of contact force: (a) non-tensile support; (b)tensile bearing.

Fig 11 displays contour maps illustrating the vertical contact force between the pier and the beam as it varies based on excitation period and amplitude. Fig 7a depicts the peak pressure at the base of the pier when subjected to a non-tensile-resistant bearing condition, while Fig 7b illustrates the peak pressure under a tensile-resistant bearing condition. A comparative analysis of these maps reveals consistent patterns in the relationship between the maximum pressure at the base of the pier and both excitation period and amplitude under the two bearing conditions. It is noteworthy that the tensile-resistant bearing condition exhibits a tensile force response within the separation interval. Moreover, the maximum pressure at the base of the pier is slightly higher under the non-tensile-resistant bearing condition compared to the tensile-resistant bearing condition. Throughout the entire calculation period, both scenarios demonstrate peak pressures approximately 3.6 times the static contact force. This suggests that the separation-collision process within the structural system elevates the pressure at the base of the pier to a certain degree.

**Fig 12:**
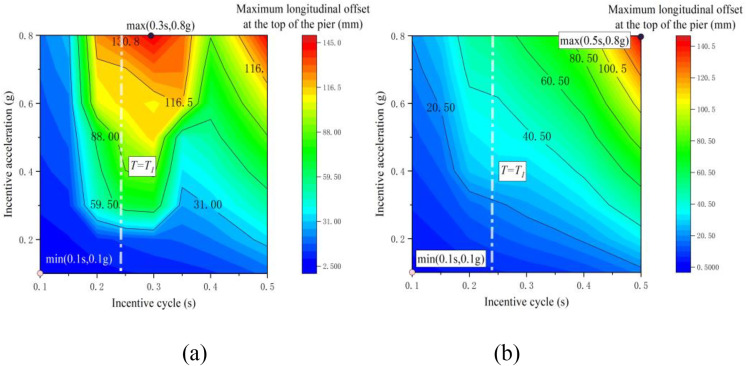
Cloud map of offset values: (a) tensile support; (b) non-tensile bearing.

Contour maps in Fig 12 illustrate the longitudinal displacement at the pier’s top concerning excitation period and amplitude. Fig 12a displays the contours for the non-tension-bearing support condition, while Fig 12b exhibits the results for the tension-bearing support condition. Analysis reveals that, irrespective of the support conditions, the longitudinal displacement at the pier’s top increases consistently with excitation amplitude. Particularly, under tension-bearing support, the maximum longitudinal displacement rises steadily with excitation period, peaking at 141.68 mm at (0.5 s, 0.8 g). Conversely, under non-tension-bearing support, vertical separation leads to a loss of horizontal constraint at the bearing, resulting in a prominent peak near the vertical first natural period (T = T1); the maximum displacement reaches 145.24 mm at (0.3 s, 0.8 g). This disparity underscores the substantial impact of the bearing’s tension-bearing capacity and the vertical separation phenomenon on the longitudinal displacement response at the pier’s top.

**Fig 13:**
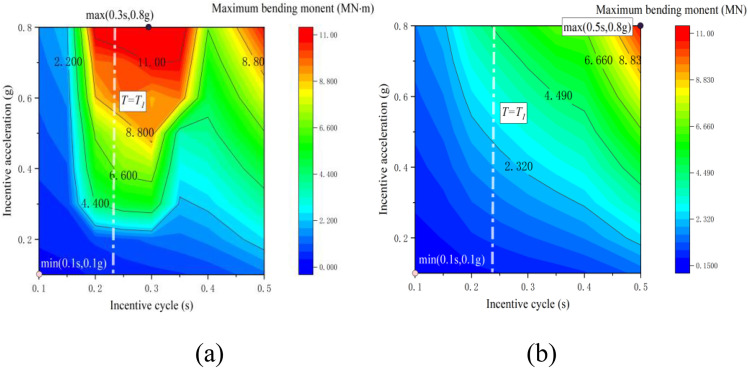
Cloud map of bending moment: (a) tensile support; (b) non-tensile bearing.

**Fig 14:**
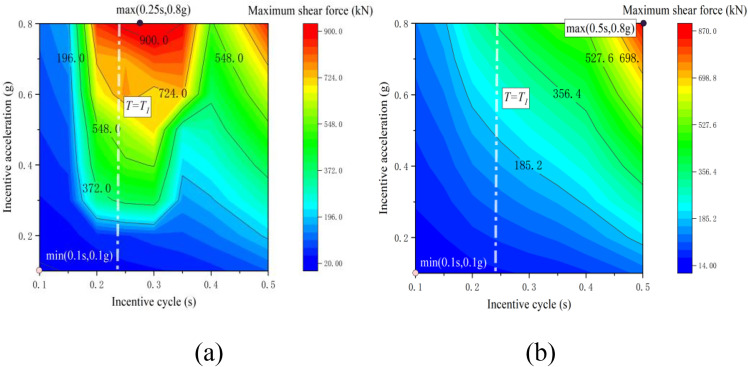
Cloud map of shear force: (a) tensile support; (b) non-tensile bearing.

Figs 13 and 14 present contour maps depicting the maximum bending moment and maximum shear force at the base of the bridge abutment under varying support conditions. The characteristic cloud patterns of both maps align closely with the distribution characteristics of the longitudinal offset extreme values observed at the top of the abutment. In the case of non-tension bearing, the extreme value of the bending moment at the bottom of the abutment occurs at (0.3s, 0.8g), reaching a maximum of 11.6 MNm. The extreme value of the shear force at this location is found at (0.25s, 0.8g), with a maximum of 908.6 kN. Conversely, for the tension bearing scenario, both the extreme values of bending moment and shear force at the bottom of the abutment are concentrated at (0.5s, 0.8g), with maximum values of 10.8 MNm and 860.2 kN, respectively.

## 5 Structural responses under actual earthquake actions

Previous studies used harmonic excitation to determine the influence of period and amplitude on the dynamic response. In this section, actual seismic waves are selected for analysis.

### 5.1 Selection of seismic waves

**Fig 15:**
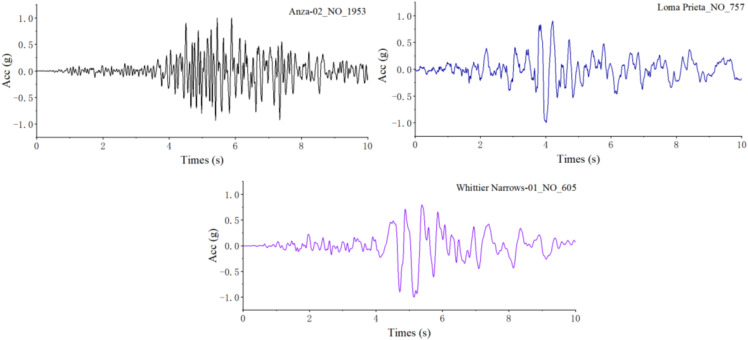
Actual seismic waves.

In this study, three seismic waves were selected as excitation inputs for analysis. The natural periods of the selected seismic waves are mainly concentrated in the range of Tg = 0.2 s – 0.6 s. Fig 15 shows the time – history response curves of the seismic waves after normalization.

Table 2 lists the specific parameters (including key indicators such as time of occurrence, moment magnitude, fault mechanism, and horizontal PGA) of the Anza, Loma Prieta, and Whittier Narrows seismic waves used in this study.

**Table 2:** Earthquake information data.

Earthquake name	Occurrence time (year)	Moment magnitude (Mw)	Fault distance (km)	Horizontal PGA (g)	Vertical PGA (g)	Horizontal PGV (cm/s)	Vertical PGV(cm/s)
Anza	1980	5.2	17.3	0.122	0.08	5.2	3
Loma Prieta	1989	6.93	7.6	0.57	0.37	95	65
Whittier Narrows	1987	5.99	41.2	0.527	0.227	24.2	6.2

Table 3 lists the first five orders of excitation frequencies of each seismic wave. To comprehensively investigate the dynamic response characteristics of the bridge structure under the excitation of actual seismic waves, it is necessary to perform amplitude modulation on the three seismic waves in Fig 9 according to the response spectrum, so that they better conform to the intensity characteristics of actual seismic actions and provide a more reasonable excitation input for the subsequent dynamic response analysis.

**Table 3:** The first five-order excitation frequencies.

Order Seismic waves	1^st^	2^nd^	3^rd^	4^st^	5^st^
Anza	34.213	33.145	39.172	26.455	35.765
Loma Prieta	15.724	17.496	15.246	13.738	19.258
Whittier Narrows	10.412	10.559	8.796	9.127	9.938

### 5.2 Internal forces at the bottom of the pier

**Fig 16:**
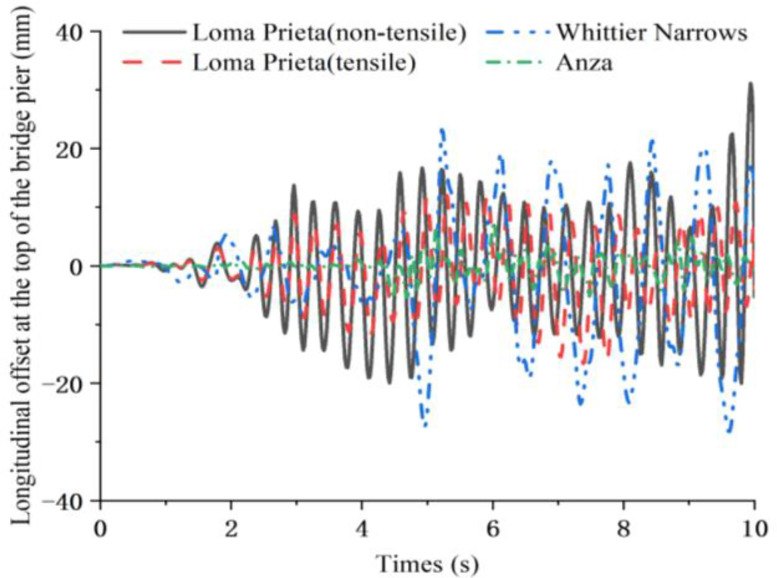
Longitudinal response at the top of the bridge pier.

Fig 16 shows the variation of the longitudinal offset at the top of the bridge pier under different seismic wave excitations: The characteristic frequency of the Anza seismic wave is far from the natural frequency of the bridge’s longitudinal direction, and the dynamic response of the longitudinal offset at the top of the bridge pier is relatively small. The characteristic frequency of the Whittier Narrows seismic wave is close to the first-order natural frequency in the longitudinal direction of the bridge, resulting in a significantly larger extreme value of the longitudinal offset at the top of the bridge pier. The characteristic frequency of Loma Prieta seismic waves is close to the first-order natural frequency in the vertical direction of the bridge, and the extreme value of the longitudinal offset response at the top of the bridge pier is significantly smaller than the result under the excitation of Whittier Narrows seismic waves. It should be particularly noted that when vertical excitation causes vertical separation of the structure, the extreme value of longitudinal offset at the top of the bridge pier will increase significantly (from 16.6 mm to 30 mm).

**Fig 17:**
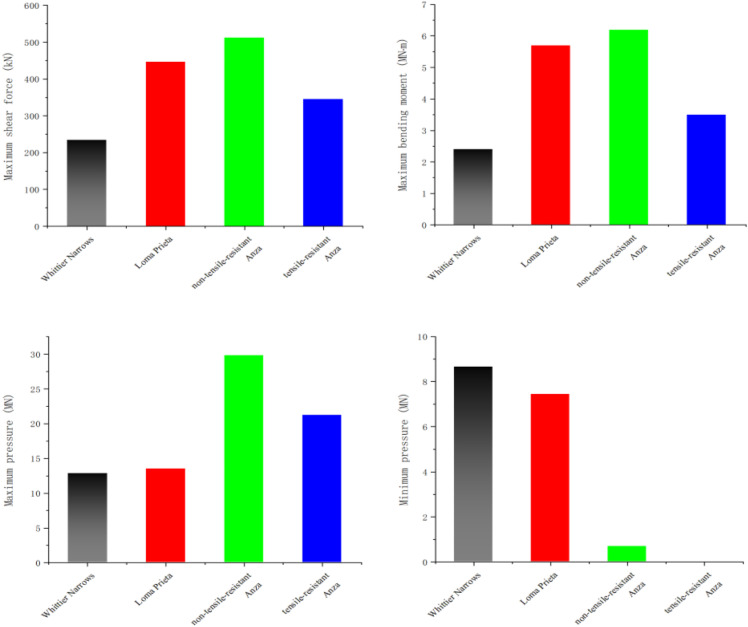
Internal forces at the pier base.

Fig 17 illustrates the maximum internal force characteristics at the base of the pier when subjected to three distinct seismic waves. Discrepancies can be elucidated by examining the correlation between the characteristic frequency of the seismic waves and the natural frequency of the bridge. In the case of the Anza earthquake, its characteristic frequency closely aligns with the first-order vertical natural frequency of the bridge. Consequently, the response disparities between non-tensile and tensile bearings are separately assessed under this seismic wave excitation. The characteristic frequency of the Whittier Narrows earthquake deviates significantly from the first-order longitudinal and vertical natural frequencies of the bridge. Consequently, both the vertical and longitudinal dynamic responses of the structure remain subdued, resulting in relatively minor maximum pressure, bending moment, and shear force at the base of the pier. In contrast, for the Loma Prieta earthquake, while its characteristic frequency approximates the first-order longitudinal natural frequency of the bridge, the vertical dynamic response is limited. However, the horizontal dynamic response escalates notably, leading to a substantial increase in the maximum shear force and bending moment at the base of the pier compared to the Whittier Narrows earthquake.

Further analysis of the impact of the Anza earthquake reveals that due to its close proximity to the first-order vertical natural frequency of the bridge, the maximum pressure at the pier bottom significantly increases compared to the effects of other seismic waves, showing an increase of 1.5 to 2 times. Specifically, the utilization of tensile-resistant bearings results in the generation of a certain level of tensile force at the base of the pier. Conversely, structures equipped with non-tensile-resistant bearings experience a separation of the main girder from the pier during seismic activity. This separation leads to the failure of the bearing’s horizontal limiting function, subsequently amplifying the pier’s horizontal dynamic response. This amplification is evidenced by a notable rise in both the maximum bending moment and maximum shear force at the pier bottom.

Equation 25 gives the allowable internal force values at the bottom of the pier, and the parameters in it refer to the specification [29]. It can be seen that each allowable value of the pier is related not only to the structural parameters but also to whether the pier is in tension and its longitudinal deformation.


$$\begin{array}{cc} \text{Allowable pressure} & \gamma_{\text{0}}N_{d}\leq n_{u}Af_{cd}\\ \text{Allowable shear} & \begin{array}{c} V_{c}\leq \phi (V_{c}+V_{s})\\ V_{c}=0.1\upsilon_{c}A_{e} \end{array}\\ \text{Allowable bending moment} & M_{ud}=\frac{\text{2}}{\text{3}}f_{cd}A_{r}\frac{{sin}^{\text{3}}\pi \alpha }{\pi }+f_{sd}A_{s}r_{s}\frac{sin\;\pi \alpha +sin\;\pi \alpha_{t}}{\pi } \end{array}$$ (25)

**Fig 18:**
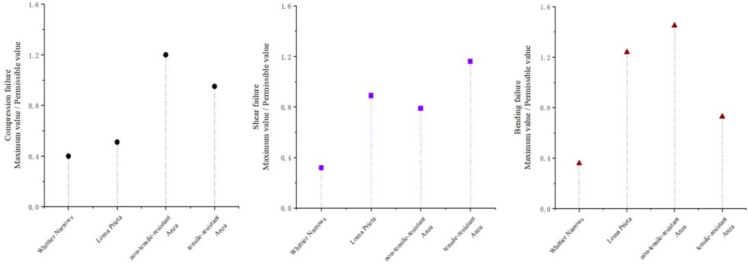
Shows the failure situation at the pier base.

Fig 18 illustrates the damage at the base of the abutment under three distinct types of earthquakes, as per the specified requirements. The quantitative indicator employed for the safety evaluation is the “ratio of the maximum value to the permissible value.” For the Whittier Channel seismic waves, the maximum-to-permissible ratios for all metrics related to the abutment foundations are below 0.5, significantly under the safety threshold of 1.0. This finding indicates that the structure remains within a safe zone, attributable to its characteristic period being substantially distant from the first-order intrinsic period of the bridge in both vertical and longitudinal directions. Conversely, under the influence of the Loma Prieta seismic wave, the characteristic period approaches the longitudinal first-order intrinsic cycle of the bridge, resulting in structural resonance. This resonance induces considerable longitudinal deformation in the pier body, leading to buckling damage. From a quantitative perspective, the maximum shear force to permissible value ratio is 0.89, nearing the critical damage level. Additionally, the maximum bending moment to permissible value ratio is 1.24, exceeding the safety threshold, which results in bending damage and significantly diminishes the overall safety margin.

**Fig 19:**
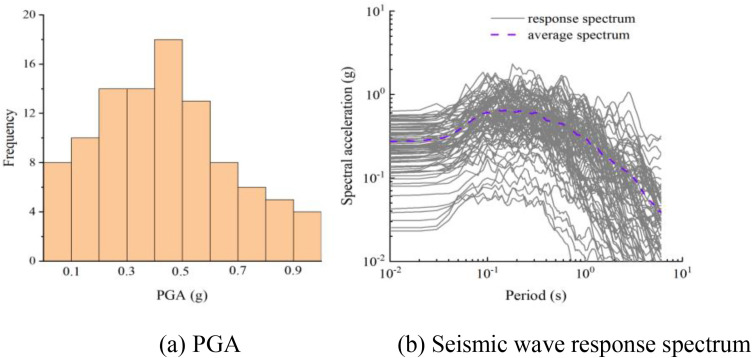
Seismic wave information.

The damage characteristics resulting from the Anza earthquake exhibited considerable variation based on the type of bearing employed. In structures utilizing tensile bearings, these components effectively mitigated lateral deformation by applying restraints. However, the transmitted tensile forces generated tensile stresses at the abutment foundations, resulting in intervals where the pressure reached zero. This condition led to a marked reduction in the allowable shear capacity of the abutments. Quantitative analysis revealed that the maximum shear force to allowable value ratio was 1.14, surpassing the safety threshold and resulting in shear damage to the bridge pier. In contrast, the maximum ratios for bending moment and pressure remained below 0.9, indicating that, although near the critical threshold, corresponding damage did not occur. Consequently, shear damage predominated, while the bending and compression states approached critical damage levels. Conversely, structures equipped with non-tensile bearings did not exhibit tensile phenomena at the abutments. However, the separation of the girder from the abutment amplified lateral deformation, indirectly lowering the permissible pressure threshold, while eccentric collisions exacerbated the overall damage. Quantitative analysis indicated that the maximum allowable values of pressure and bending moments for this type of structure were 1.21 and 1.43, respectively, both of which exceeded the safety thresholds, resulting in compression and bending damage. In contrast, the maximum allowable value of shear was 0.79, which did not reach the damage threshold; consequently, no shear damage occurred Fig 19.

### 5.3 Analysis of the vulnerability of bridge piers

To fully consider the randomness of the seismic spectrum, with peak groundacceleration (PGA) as the indicator of ground motion intensity, 100 seismic waves were selected from the strong ground motion database of the Pacific Seismic Engineering Research Center, as shown in Fig 4.

In the previous work of this study, a systematic analysis has been conducted on the main failure modes of bridge piers, such as compression, shear and bending. The following section will focus on the research of the vulnerability of bridge piers. Due to the current lack of clear and standardized damage indicators for compression failure, and the fact that this failure mode mainly occurs in the case of separation collision of non-tensile bearings under specific frequency excitation, and this type of compression failure is closely related to bending failure, the vulnerability analysis in this paper will mainly focus on bending and shear failure modes.

Table 4 [30] presents the shear and bending damage indicators of bridge piers. For the damage indicators in Table 4, among them, *V*_*d*_ (*V*_*k*_*, V*_*m*_) are the bearing capacities of the inclined sections of the components calculated respectively based on the design values of material strength (standard values, average values). $\phi_{y},\;\phi_{I0},\;\phi_{p},\;\phi_{u}$ represents the first to fourth curvatures of the bridge piers respectively. The calculation formulas can be found in formulas 26–28.

**Table 4:** Bridge Pier Damage Indicators.

Damage situation Component names	Minor damage	moderate damage	Severe damage	complete damage
Pier bending	$V_{d}$	$0.5(V_{d}+V_{k})$	$0.5(V_{k}+V_{m})$	$V_{m}$
Pier shearing	$\phi_{y}$	$\phi_{I0}$	$\phi_{p}$	$\phi_{u}$

For the bending of bridge piers, the yield curvature is:


$$\phi_{y}=1.957\varepsilon_{y}/H$$ (26)

The second and third degrees of curvature are respectively:


$$\phi_{I0}=1.5\theta_{y}\text{,}\;\;\phi_{p}=\phi_{y}+0.3\left(\phi_{u}-\phi_{y}\right)$$ (27)

The limiting curvature is:


$$\phi_{u}D=min\left\{\begin{array}{c} @c@(2.826\times 10^{-3}+6.850\varepsilon_{cu})-(8.575\times 10^{-3}+18.638\varepsilon_{cu})\left(\frac{P}{f_{ck}A_{g}}\right)\\ (1.635\times 10^{-3}+1.179\varepsilon_{s})-(28.739\times \varepsilon_{s}^{\text{2}}+0.656\varepsilon_{s}+0.010)\left(\frac{P}{f_{ck}A_{g}}\right) \end{array}\right\}$$ (28)

**Fig 20:**
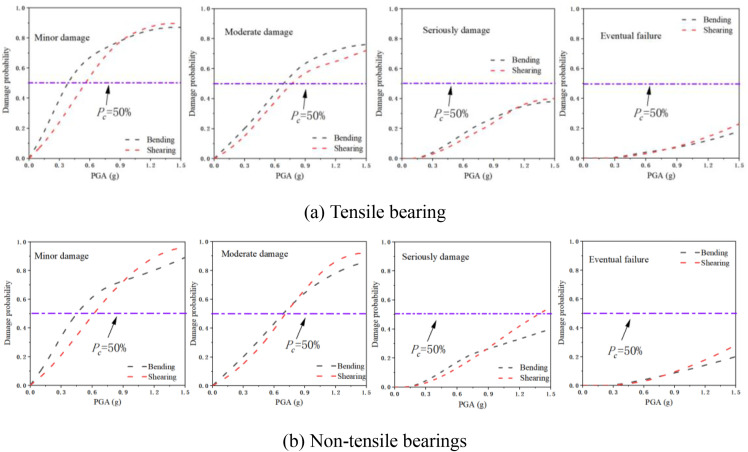
The vulnerability curves of the bottom of bridge piers under different conditions.

Fig 20 illustrates the vulnerability of bridge piers with various support types. For tensile bearings, the probabilities of bending and shear failure during the minor and moderate damage stages increase monotonically with the peak ground acceleration (PGA). Furthermore, under identical PGA conditions, the risk of bending failure at the base consistently surpasses that of shear failure. In the severe damage and complete damage stages, the probability of bending failure remains higher than that of shear failure at relatively low PGAs. However, as the PGA continues to rise, the likelihood of shear failure gradually overtakes that of bending failure. In the case of non-tensile bearings, the failure characteristics during the minor damage stage resemble those of tensile bearings. At low PGAs, the probability of bending failure is predominant; as the PGA increases, the risk of shear failure becomes more significant. During the moderate and severe failure stages, the probabilities of bending and shear failure alternately rise with increasing PGA. In the complete damage stage, when the PGA exceeds 1.0g, the failure probabilities for both types of failure increase. Notably, at PGA = 1.5 mm, the probability of bending failure is 40 percentage points higher than that of shear failure.

A comparison of the two types of bearings reveals that their bending and shear failure characteristics are similar during the minor and moderate failure stages. However, in the severe and complete failure stages, the shear failure probability of the tensile bearing approaches that of the non-tensile bearing, while the risk of bending failure is lower than that of the non-tensile bearing, with the maximum difference reaching 65% of the bending failure probability of the non-tensile bearing.

For the two modes of bending failure and shear failure of bridge piers, the two together constitute the overall failure form of bridge piers, and logically they are in a series relationship. Based on the reliability analysis principle of the series system, the failure probability of bridge piers can be solved through the failure probability formula of the series system:


$$P_{S}=P(G_{B}\leq 0⋃G_{S}\leq 0)$$ (29)

Among them, $P_{S}$ represents the overall failure probability of the bridge pier, $G_{B}$ and $G_{S}$ are the function function of the bridge pier subjected to bending and shear function, the function function value is less than or equal to 0, corresponding to the bending damage or shear damage occurs.

**Fig 21:**
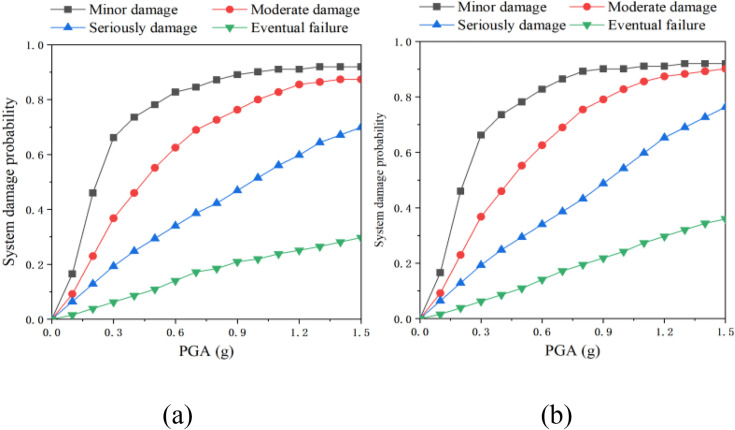
Comparison of vulnerability of bridge piers: (a) Tensile strength; (b) Non-tensile.

The analysis results (Fig 21) indicate that the piers associated with the two types of bearings did not exhibit any separation phenomenon under seismic action when the peak ground acceleration (PGA) was below 0.4g, resulting in a similar risk of damage to the piers. As the PGA amplitude increased, the non-tensile bearing experienced separation from the abutment, leading to a continuous increase in longitudinal offset. This phenomenon directly contributed to a heightened probability of damage to the bridge abutment. In terms of damage severity, the risk of slight damage to the abutment for both types of supports was essentially equivalent. However, as the degree of damage intensified, the disparity in damage probabilities between the two types of bearings gradually widened. Upon reaching the complete damage stage, the damage probability for the bridge pier associated with the non-tensile bearing was 0.35, significantly exceeding the damage probability of 0.29 for the tensile bearing, representing a difference of 21%.

## 6 Influence of pier height on structural damage

**Fig 22:**
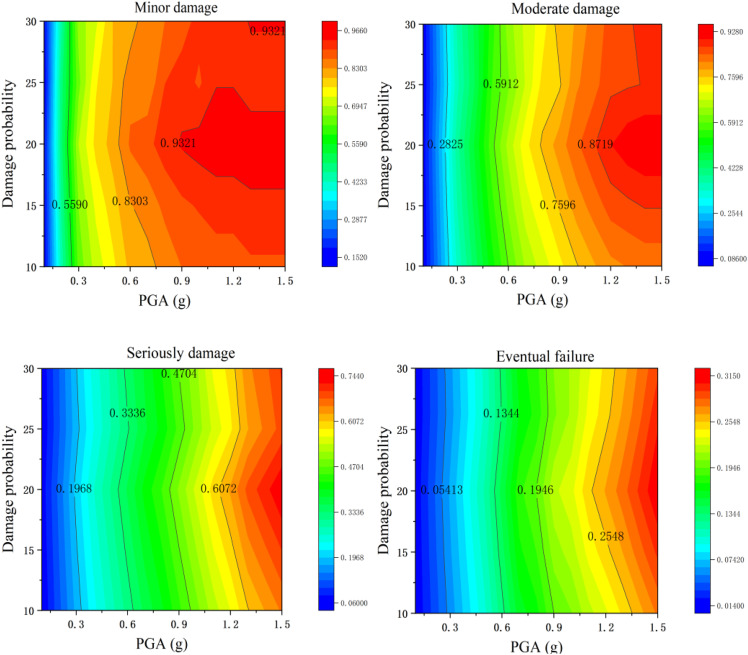
Probability of Vulnerability of Bridge Piers at Different Heights (Tensile Bearings).

Through the quantitative analysis of the relationship curve of “structural damage probability with tensile bearings - pier height” in Fig 22, it can be known that within the range of pier height parameters involved in this study, as the pier height increases, the structural damage probability does not show a significant changing trend, and the overall fluctuation range is relatively small.

**Fig 23:**
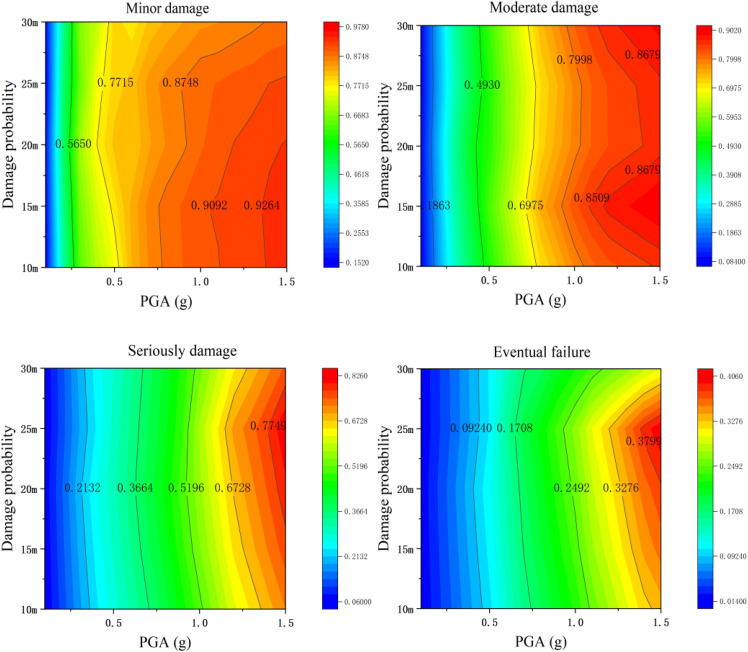
Vulnerability Probability of Bridge Piers at Different Heights (Non-tensile bearings).

Specifically, further observation of the curve characteristics reveals that only when the height of the bridge pier is 20 meters does the probability of structural damage show a weak peak. However, in the remaining height intervals (such as those below 20m or above 20m), the numerical distribution of the probability of structural damage is relatively concentrated, and no significant difference caused by changes in the height of the bridge piers is observed Fig 23.

Under non-tensile bearings, the dynamic response of bridge piers changes due to the separation effect between the bearing and the pier, and their vulnerability varies significantly with the height (H) of the pier, in contrast to the stable performance of tensile bearings. The differences in the influence of the two types of supports show phased characteristics: In the first two stages of damage (minor and moderate damage), there is no significant difference in the influence between the two, as the horizontal excitation dominates the damage at this stage and the vertical separation effect is covered. Entering the latter two stages of damage (severe and complete destruction), when the peak ground acceleration (PGA) exceeds 0.8g, the difference becomes apparent – the peak probability of damage to the tensile bearing is H = 20m; The peak value of the non-tensile bearing shifted to H = 25m, and the probability of failure was significantly higher. Due to the change in the longitudinal frequency of the bridge pier before and after separation, the extreme value of the offset increased, and the probability of severe damage increased by 12% and the probability of complete failure increased by 21%. When H = 30m, the difference between the two types of bearings Narrows. As the height of the bridge pier increases, the vertical flexibility is enhanced, the probability of separation between the bearing and the bridge pier decreases, and the damage amplification effect of the non-tensile bearing is weakened.

## 7 Dicussion

This study builds a numerical model based on the OpenSees platform, focusing on the influence mechanism of parameters related to structural separation under seismic excitation. By inputting multiple sets of actual seismic waves, the excitation evolution process of bridge pier failure and the dynamic response characteristics of bridge piers of different heights under different bearing conditions were systematically explored, providing a fundamental support for understanding the action path of structural separation on bridge pier failure.

1. From the perspective of the correlation law between excitation parameters and structural responses, the matching degree between the seismic period and the vertical first-order natural vibration period of the bridge is a key factor influencing the mechanical behavior of the bearing. When the two are close, the maximum axial pressure of the support will increase exponentially with the amplitude. The underlying reason is the continuous accumulation of energy caused by the resonance effect. During the stage when the structure has not separated, the peak axial pressure always shows a linear relationship with the amplitude. After the structure undergoes separation, the sensitivity of collision force to amplitude will significantly decrease. The core reason is that the separation gap alters the original contact force transmission mechanism of the structure, thereby reducing the transmission efficiency of amplitude changes to the collision effect. In addition, the vertical collision response of the middle span in equal-span continuous beam Bridges is superior to that of the side span. This is because there are differences in the constraint conditions and force distribution between the middle span and the side span: the more uniform force state of the middle span can effectively reduce the local collision effect.
2. For non-tensile bearings, when the seismic period is close to the first-order vertical natural vibration period of the bridge, it is easy to cause vertical separation between the main beam and the bearing. The failure of the horizontal constraint of the bearing will further amplify the longitudinal dynamic response of the bridge pier, ultimately leading to a significant increase in the maximum bending moment and maximum shear force at the bottom of the bridge pier.
3. The correspondence between the characteristic frequencies of seismic waves and the natural frequencies of Bridges directly affects the structural response intensity. For instance, in the Anzha earthquake, a specific frequency arrangement significantly increased the pressure on the pier foundation, confirming the dynamic amplification effect caused by frequency matching. The regulatory effect of bearing types on the response of bridge piers is also very significant. Non-tensile bearings can cause beam-pier separation and break the original constraint system of the structure, thereby amplifying the horizontal dynamic response of the bridge pier. This mechanism provides an important reference for bearing selection and seismic design of the structure. The differences in the failure modes of bridge piers under different seismic waves further reflect the influence of seismic motion characteristics. For instance, the structure remains stable under the Wheatier Strait earthquake, while the Lomo Pretta earthquake is prone to induce bending failure of bridge piers. In the Anza earthquake, tensile bearings are prone to cause shear-bending combined failure of bridge piers, while non-tensile bearings trigger compression-bending failure. These differences are all closely related to the spectral characteristics, amplitude distribution and duration of seismic waves in various regions.
4. The analysis of the vulnerability of bridge piers shows that the differences in vulnerability between the two types of bearings mainly occur in the high-damage stage and the high-PGA working condition: for tensile bearings, the risk of bending failure is always higher than that of shear failure in the mild to moderate damage stage. Only when the PGA expands to the severe damage stage does the risk of shear damage exceed that of bending. The risk changes of non-tensile bearings are more complex. During the moderate and severe failure stages, there will be an alternating increase in the risks of bending and shearing. Moreover, in the complete failure stage (when PGA = 1.5g), the risk of bending failure is 40 percentage points higher than that of shearing. The essence of the above-mentioned differences stems from the distinct constraint characteristics of the two types of bearings – non-tensile bearings are prone to causing beam-pier separation, and by regulating the dynamic response of the structure, the failure risk law shows a significant difference from that of tensile bearings.
5. The probability of bridge pier damage under tensile bearings does not change significantly with the increase in height, only showing a weak peak at 20m, and the overall fluctuation is relatively small. However, due to the separation effect between the bearing and the pier of the non-tensile bearing, its vulnerability varies significantly with height. The difference in influence between the two is phased – in the first two damage stages, the damage is dominated by horizontal excitation, and there is no obvious difference between the two types of bearings. But in the last two damage stages (PGA > 0.8g), the difference becomes apparent. The peak damage probability of the tensile bearing is 20m. The peak of the non-tensile bearing shifts to 25m and the probability of failure is significantly higher (the probability of severe damage increases by 12% and the probability of complete failure increases by 21%). When the height increases to 30m, due to the enhanced vertical flexibility of the bridge pier, the probability of separation between the bearing and the bridge pier decreases, and the damage amplification effect of the non-tensile bearing is weakened, narrowing the difference between the two types of bearings.

Based on the correlation analysis of bearing types, pier heights and seismic excitation in this study, in the future, the focus can be further deepened in three aspects: First, the parameter design of bearing materials, which needs to explore the quantitative correlation between the mechanical properties of materials and the beam-pier separation threshold, providing a parameter basis for the anti-separation optimization of non-tensile bearings; The second is the influence of soil parameters. It is necessary to focus on analyzing the regulatory effect of the stiffness and damping characteristics of the foundation on the structural constraint state, and clarify how they change the separation probability and dynamic response of the bearing and the pier. Thirdly, for the design of limit devices under separable conditions, it is necessary to combine the separation damage mechanism revealed in this study, optimize parameters such as the stiffness and clearance of the device, and while allowing reasonable separation, suppress the damage to bridge piers caused by excessive collision, ultimately enhancing the seismic safety of the structure.

## 8 Conclusion

This study constructed a numerical model based on the OpenSees platform. By inputting multiple sets of actual seismic waves, it focused on the influence mechanism of parameters related to structural separation under seismic excitation, explored the evolution process of bridge pier failure excitation and the dynamic response characteristics of bridge piers at different heights under different bearing conditions, providing a fundamental support for understanding the action path of structural separation on bridge pier failure.

1. The excitation parameters are significantly correlated with the structural response: when the seismic period matches the first-order vertical natural vibration period of the bridge, the maximum axial pressure of the bearing increases exponentially with the amplitude. After structural separation, the sensitivity of collision force to amplitude decreases, and the vertical collision response of the middle span in equal-span continuous beam Bridges is better than that of the side span. Non-tensile bearings are prone to vertical separation of beams and piers due to periodic matching, amplifying the longitudinal dynamic response of bridge piers and the internal forces at the bottom.
2. The type of bearing and the height of the bridge pier jointly affect the vulnerability: Under tensile bearings, the probability of bridge pier damage fluctuates little with height. For non-tensile bearings, the damage probability is prone to change in stages with height due to the separation effect (the difference is significant when PGA > 0.8g, and the probability of complete failure is 21% higher). The differences in the failure risks of the two types of bearings are mainly concentrated in high-damage and high-PGA conditions, and the non-tensile bearings have a higher risk of bending failure.
3. Seismic motion characteristics regulate the response and failure mode of bridge piers: The matching of the characteristic frequency of seismic waves with the natural frequency of the bridge will amplify the structural response (such as the Anza earthquake). Different seismic waves, due to differences in spectrum, amplitude, etc., cause bridge piers to present different failure modes such as bending and shear-bending. The type of bearing further affects the differentiation of failure modes.
4. Future research can be deepened in three aspects: First, explore the quantitative correlation between the mechanical properties of bearing materials and the beam-pier separation threshold; The second is to analyze the influence of soil parameters (foundation stiffness, damping) on the separation of bearings and piers and dynamic response; Thirdly, in combination with the separation damage mechanism, optimize the stiffness and clearance parameters of the lower limit device for separable working conditions.
